# Study protocol on prevalence of non-exudative macular neovascularisation and its contribution to prediction of exudation in fellow eyes with unilateral exudative AMD (EYE-NEON)

**DOI:** 10.1038/s41433-023-02460-9

**Published:** 2023-03-07

**Authors:** Sridevi Thottarath, Shruti Chandra, Sarega Gurudas, Wei-Shan Tsai, Andrea Giani, Eduard De Cock, Taffeta Ching Ning Yamaguchi, Sobha Sivaprasad, EYENEON Study Group, Ben Burton, Geeta Menon, Manju Chandran, Ian Pearce, Savitha Madusudhan, Anna Grabowska, Faruque Ghanchi, James Talks, Richard Gale, Martin Mckibbin, Ajay Kotagiri, Niro Narendran, Afsar Jafree, Saad Younis, Claire Bailey, Priya Prakash, Christiana Dinah, Nitin Gupta, Louise Downey, Andrew Lotery, Paritosh Shah, Romi Chhabra, Narendra Dhingra, Indra Dias, Mary Freeman, Daren Hanumanthudu, Asma Burale, Radha Ramakrishnan

**Affiliations:** 1 NIHR Moorfields Clinical Research Facility and Biomedical Research Centre, Moorfields Eye Hospital, London, UK; 2 Institute of Ophthalmology, University College London, London, UK; 3 Boehringer Ingelheim International GmbH, Ingelheim am Rhein, Germany; 4 James Paget University Hospital NHS Foundation Trust, Great Yarmouth, UK; 5 Frimley Health NHS Foundation Trust, Camberley, UK; 6 Liverpool University Hospital NHS Foundation Trust, Liverpool, UK; 7 Kings College Hospital NHS Foundation Trust, London, UK; 8 Bradford Teaching Hospital NHS Foundation Trust, Bradford, UK; 9 The Newcastle upon Tyne Hospital NHS Foundation Trust, Newcastle upon Tyne, UK; 10 York Teaching Hospital NHS Foundation Trust, North Yorkshire, UK; 11 Leeds Teaching Hospital NHS Foundation Trust, Leeds, UK; 12 South Tyneside and Sunderland NHS Foundation Trust, Sunderland, UK; 13 The Royal Wolverhampton NHS Foundation Trust, Wolverhampton, UK; 14 East Kent Hospital NHS Foundation Trust, Canterbury, UK; 15 Imperial College NHS Foundation Trust, London, UK; 16 University Hospital Bristol and Weston NHS Foundation Trust, Bristol, UK; 17 The Princess Alexandra Hospital NHS Trust, Harlow, UK; 18 London North West University Healthcare NHS Trust, Harrow, UK; 19 West Suffolk NHS Foundation Trust, Bury St Edmunds, UK; 20 Hull University Teaching Hospital NHS Foundation Trust, Kingston upon Hull, UK; 21 University Hospital Southampton NHS Foundation Trust, Southampton, UK; 22 Yeovil District Hospital NHS Foundation Trust, Yeovil, UK; 23 Manchester University NHS Foundation Trust, Manchester, UK; 24 Mid Yorkshire Hospitals NHS Foundation Trust, Wakefield, UK; 25 Calderdale and Huddersfield NHS Foundation Trust, Huddersfield, UK; 26 Sheffield Teaching Hospital NHS Foundation Trust, Sheffield, UK; 27 Royal Free London NHS Foundation Trust, London, UK

**Keywords:** Predictive markers, Macular degeneration

## Abstract

**Purpose:**

Fellow eyes of patients with unilateral neovascular age-related macular degeneration (nAMD) are at risk of developing macular neovascularisation (MNV). These eyes may first develop subclinical non-exudative MNV (neMNV) before they leak to form exudative MNV (eMNV). The EYE NEON study is a 2-year study aimed at estimating the prevalence and incidence of neMNV and evaluating its role as a predictor for conversion to neovascular AMD.

**Methods:**

EYE NEON is a multicentre study that will run in retinal clinics across 25 National Health Service with the aim to recruit 800 patients with new onset nAMD in the first eye. The fellow-eye with no evidence of nAMD at baseline will be the study eye. All study eyes will have OCT and OCTA done at first and second year following first anti-VEGF treatment to the first eye (non-study eye), with new onset nAMD. We will estimate the prevalence and incidence of neMNV over 2 years, rate of conversion from neMNV to eMNV and numbers initiated on treatment for neovascular AMD in the study eye will be reported. Predictive models of conversion including neMNV with other demographic and imaging parameters will be developed.

**Conclusion:**

The study design with proposed target sample size is sufficient to evaluate the retinal imaging characteristics of the study eyes with and without neMNV and develop predictive models to inform risk of conversion to nAMD.

## Introduction

Active new onset neovascular age-related macular degeneration (nAMD) is characterized by the presence of macular neovascularisation (MNV) that leak fluid and/or blood. Exudation from MNV responds to anti-vascular endothelial growth factor (anti-VEGF) agents [1]. Nevertheless, prevention of conversion of fellow eyes with high-risk intermediate AMD to neovascular AMD with quarterly intravitreal injections of anti-VEGF agents has failed to show any benefit [2, 3]. On average, the risk of development of MNV in fellow eye increases at the rate of about 10% per year [4–6]. However, there is significant variations in the incidence of MNV in fellow eyes [7–10]. Therefore, intermediate AMD in the fellow eye per se is not sufficient to predict conversion. One marker of conversion identified on optical coherence tomography (OCT) is the presence or new onset double layer sign (DLS) defined as a localized separation of the retinal pigment epithelium from the Bruch’s membrane [11–13]. Some of these DLS fit the criterion of shallow, irregular retinal pigment epithelial elevation (SIRE) (defined as greatest transverse linear dimension of 1000μm or more, an irregular RPE layer with a height of predominantly less than 100 μm, and a nonhomogeneous internal reflectivity [14, 15]. In addition, some of these DLS may harbor subclinical non-exudative MNV (neMNV) detected by OCT-angiography (OCTA) and this sign may more accurately predict conversion to neovascular AMD compared to DLS without neMNV [16, 17]. The incidence and prevalence of neMNV is unclear with reports from studies with small sample sizes indicating ranges from 6 to 60% [17–22] at varying timepoints. It is also unclear whether any morphological features of neMNV may further aid the prediction of conversion to eMNV. Risk models for conversion to neovascular AMD need to be developed.

## Objective

The primary objective of the study is to quantify the prevalence of neMNV in fellow eyes (study eyes) of patients with new onset unilateral neovascular AMD (non-study eye). Secondary objectives include estimating the incidence of neMNV over 2 years in study eyes, descriptive analysis of eyes with and without neMNV and developing a risk model for conversion to neovascular AMD incorporating both demographic and retinal imaging features over 2 years.

## Study design

This multi-center cohort study was approved by London-City and East research ethics committee (REC reference 20/PR/0897) and will recruit ~800 patients with new onset unilateral nAMD that have been initiated on anti-VEGF therapy. This will include a retrospective cohort who were initiated on first anti-VEGF therapy in non-study eye not more than 15 months prior to date of consent to this study. The prospective cohort will include patients with new onset unilateral nAMD who will provide consent on date of first anti-VEGF agent. In both cohorts, there should be no evidence of any exudation due to nAMD in the fellow (study) eyes. They will be followed up for 2 years. Year 1 and 2 will be calculated from date of first anti-VEGF injection received in non-study eye.

## Study population

It is anticipated that the study will run across 25 different National Health Service sites in the UK. Patients are eligible for the study if they are aged between ≥50 years and ≤100 years and have a diagnosis of unilateral treatment naïve exudative neovascular AMD at baseline and initiated on anti-VEGF therapy. The fellow eye is the study eye and may have any stage of non-neovascular AMD (early or intermediate AMD or geographic atrophy). The study eye had to have adequate media clarity, pupillary dilation, and patient cooperation for capturing OCT and OCTA imaging. The exclusion criteria included treatment initiation for unilateral neovascular AMD more than 15 months prior to recruitment, advanced AMD in the study eye (geographic atrophy or neovascular AMD or any other ocular condition that, in the opinion of the investigator, might affect or alter visual acuity during the course of the study. Other exclusion criteria included any patient who has opted out of their information being used for research nationally or locally at any site, study eyes treated with anti-VEGF for other causes of MNV or patients participating in a clinical trial with an ophthalmic experimental therapy.

## Study procedures and schedule of assessments

All patients will provide consent to participate in the study. For the retrospective cohort, the baseline data and retinal images obtained during routine care will be collected and then prospectively imaged as per study protocol for their year 1 and 2 visits. The prospective cohort will have retinal images captured as per study protocol at all 3 visits. Participating sites will capture OCT and OCTA images with devices available in their clinics. The majority of the clinics have Spectralis OCT/HRA + OCT (Heidelberg Engineering, Germany), with software version 6.12.x.x). During all the study visits, OCT macula volume Scan: 20° × 20° (5.9 mm × 5.9 mm) with 97 Sections, High Speed, 15 Frames (Automated Realtime Averaging-ART) will be obtained. For OCTA, ideally two scans are preferred with one high resolution 10° scan (HR10): dense preset volume scan, 512 B-Scans: 10° × 10°, 512 Sections, 5 Frames (ART) and the other is high speed 20° scan (HS20): dense preset volume scan, 512 B-Scans: 20° × 20°, 512 Sections, 5 Frames (ART). If the patient is not compliant, at least HS 20° is required. The follow up images for year 1 and 2 must be acquired using the follow up mode. The scans should also meet the quality criteria of excellent signal strength (>30 dB) with correct positioning of the scan pattern centered at fovea. For those sites that obtain TRITON OCTA, Topcon 3D OCT-1000 or Topcon OCT-2000 series with version 11.1 or higher will be used. All the study visits required OCT 3D macula 7 × 7 mm cube scan and OCTA 6 × 6 mm cube scans.

## Data collection, export and management

Data collected from baseline visit include visual acuity for both eyes, date of first injection in non-study eye, routine demographic data of age, gender, smoking history, and OCT and OCTA images. The baseline OCT of the non-study eye was also collected to confirm evidence of neovascular AMD. If OCTA was not captured at baseline as per routine care for the retrospectively collected data, it will be done within the first year of treatment to non-study eye. Visual acuity, OCT and OCTA images at year 1 and 2 have to be captured and collected within a window of ±3 months at year 1 and 2.

All study data will be handled in accordance with the Data Protection Act 2018 and General Data Protection Regulation (GDPR). The data collection tool for this study will be through an electronic data capture (EDC) system (Playon Ltd, Bengaluru, India). All access to the EDC system is through a password-protected security system. The patient identification number (PIN) will be generated by the EDC and no patient identifiable data will be entered in the database. The retinal images from the participating sites will be transferred to Moorfields Eye through encrypted NHS mail large file transfer service. For sites that do not have access to NHS mail, data will be transferred on an encrypted USB stick via courier. The retinal images will be first reviewed for data quality and study eligibility. The images will be graded by trained retinal fellows in Moorfields using a detailed grading format. Each retinal fellow will grade 50 test images and inter-grader agreement of a random 10% of OCT and OCTA images will be reported.

## Safety reporting

All adverse events and serious adverse events related to this project in terms of image capture will be collected and reported. The OCT and OCTA are non-invasive diagnostic techniques that utilize reflected light waves. There are no known side effects or complications related to these assessments.

## Statistical considerations

Sample size calculation: due to the nature of this epidemiological study, there is no (confirmatory) hypothesis testing foreseen. The study size is based on achieving sufficient precision for prevalence of neMNV and conversion from neMNV to nAMD. The recommended number of patients is ~800 patients in total. The primary objective is to understand the prevalence of neMNV. Table 1 shows several scenarios for prevalence, ranging from 5 to 70%. While the literature is limited, we expect an overall prevalence of neMNV of 20–40%. Calculations were performed using nQuery + nTermin 4.0.

**Table 1:** Estimation of prevalence of nonexudative MNV using nQuery + nTermin4.0.

Observed prevalence (%)	*N* = 800	
	Total patients	
	Lower CI	Upper CI
5.0%	3.5%	6.5%
10.0%	7.9%	12.1%
20.0%	17.2%	22.8%
30.0%	26.8%	33.2%
40.0%	36.6%	43.4%
50.0%	46.5%	53.5%
60.0%	56.6%	63.4%
70.0%	66.8%	73.2%

For estimating conversion from neMNV to nAMD, based on assumptions of patients diagnosed with neMNV and participation rates, we expect that 150–200 patients will have follow-up data. Both scenarios are considered in Table 2. While the literature is limited, we expect an overall conversion of neMNV to nAMD of 40–60% in 24 months. Those rows are highlighted in gray. Calculations were performed using nQuery + nTermin 4.0.

**Table 2:** Confidence interval (CI) estimates for different sample sizes with conversion scenarios.

Based on the above scenarios, the recommended number of patients is about 800 in total is sufficient to achieve adequate precision for both prevalence and conversion estimates.

## Statistical analysis

At baseline, we will report the prevalence of neMNV in study eyes of patients with nAMD in the non-study eye when first initiated on anti-VEGF therapy. We will compare the correlates of having neMNV diagnosis or not in the study eye at baseline. At follow-up, we will report the percent of neMNV eyes who converted to nAMD. These will include patients who have been graded as nAMD on OCT and OCTA and those initiated on anti-VEGF treatment. Cumulative incidence will be reported and time to event may be plotted. For risk models, we will evaluate risk factors that determine conversion. Subgroup analysis may be done based on duration from diagnosis of nAMD in non-study eye, first record of neMNV and other key baseline demographic and clinical characteristics.

## Outcomes

The primary outcome is to estimate the prevalence of neMNV in the study eye of patients with a diagnosis of nAMD in the first eye on the first anti-VEGF injection visit. Secondary outcomes include reporting the incidence of neMNV over the period of 2 years; conversion rate to nAMD at 1 and 2 years with and without neMNV; demographic and baseline retinal risk factors associated with converting from neMNV (as diagnosed at baseline) to nAMD during follow-up; the diagnostic accuracy of DLS, SIRE and other imaging markers to detect neMNV; changes in visual acuity during follow-up; survival analysis of time to occurrence of DLS, conversion of DLS to neMNV and neMNV to eMNV. For predictive models for neMNV and eMNV, we will evaluate risk factors for faster/slower time to progression or conversion. Subgroup analysis may be done based on other key baseline characteristics. A detailed statistical analysis plan will be developed.

## Trial management and monitoring

The trial management group (TMG) consisting of Chief Investigator, associate principal investigator, sub-Investigators, trial manager and other delegates of the group, will oversee the setting up and conduct of the study. The TMG will regularly review quality of images to assess need for further training.

## Discussion

This study is aimed to address the gap in knowledge on the prevalence and incidence of neMNV in study (fellow eyes) of people with nAMD. As fellow eyes of unilateral nAMD are at higher risk of conversion than eyes with bilateral intermediate AMD, we included this group of patients to achieve a higher event rate. Most studies on neMNV in AMD have limited sample sizes [17–22]. The strength of this study is the large sample size allowing at least a quarter of the patients to have 2-year data to accurately provide the incidence data of neMNV in unilateral nAMD patients. Previous studies have relied on DLS or SIRE as a surrogate marker of neMNV. The use of OCTA to confirm neMNV in eyes with DLS or SIRE in this study is another strength of this study.

Our definition of conversion to eMNV includes both OCT evidence of any macular fluid as well as initiation of anti-VEGF injection in the fellow eye. This is to avoid small new pockets of subretinal fluid or tiny intraretinal cysts to be mis-represented as conversion.

We also aim to look at the diagnostic accuracy of the DLS and to provide descriptive features of a DLS that has a neMNV [4–8].

The strengths of the study are the sample size that is expected to provide sufficient event rate to develop predictive models on risk of conversion and that OCTA is used to confirm neMNV in all visits.

The main limitation of the study design is that we have only three timepoints within 2 years when OCT and OCTA will be assessed. Therefore, the exact time of conversion cannot be estimated.

## Summary

### What was known before

- Subclinical non-exudative macular neovascularisation (neMNV) detected by OCTA may be present before an eye converts to neovascular AMD. But the incidence and prevalence of neMNV is unclear. Moreover, it is also unclear whether it is the strongest predictor for conversion to neovascular AMD.

### What this study adds

- Evaluating the OCT and OCTA images captured from fellow eyes of patients over 2 years from date of first anti-VEGF injection for unilateral neovascular AMD will provide both the prevalence and incidence of neMNV and the time to conversion to eMNV. Predictive models incorporating new imaging characteristics such as neMNV may provide more information on whether neMNV is indeed a risk factor for conversion of neovascular AMD.
